# Microbial growth observation and oil displacement characteristics based on *in-situ* observation technology of reservoir pore structure

**DOI:** 10.3389/fchem.2025.1682047

**Published:** 2025-10-09

**Authors:** Wei Xu, Zhaoyun Wang, Gangzheng Sun, Xin Song, Qiongyao Chen, Caifeng Li, Sen Wang, Yingxue Hu

**Affiliations:** 1 School of Human Settlements and Civil Engineering, Xi’an Jiaotong University, Xi’an, China; 2 Institute of Petroleum Engineering, Shengli Oilfield Company, Sinopec, Dongying, China

**Keywords:** microbial enhanced oil recovery, visual microfluidic chip, growth curve, diffusion coefficient, shut-in time

## Abstract

The metabolic products of functional microbial communities used in microbial oil recovery can reduce crude oil viscosity, alter interfacial tension, and modify rock wettability, all of which significantly improve the recovery of residual oil. Therefore, it is crucial to study the mechanisms occurring at the microbe–oil–rock mineral interface in porous media. However, traditional observation techniques struggle to achieve *in-situ* monitoring, limiting progress in understanding these mechanisms. This study utilized visual microfluidic chip technology to simulate realistic reservoir pore structures and combined it with microscopic *in-situ* observation techniques to investigate the growth characteristics, diffusion patterns, and oil displacement behavior of bacteria (strain WJ-8) at the pore scale. The results showed that bacterial growth followed the Logistic model, with a rapid growth phase occurring between days 1 and 3, after which the bacteria entered a stable phase and began secreting large amounts of biosurfactants. In the arched chip structure, growth was slower at the ends and faster in the middle. *In-situ* observations revealed that at the blind ends and edges of pore throats, low flow rates and multiple attachment sites favored bacterial aggregation, enhancing biofilm stability and crude oil detachment. Conversely, within the main pore channels, high shear forces caused dispersed bacterial growth and resulted in lower oil displacement efficiency. By monitoring bacterial activation times at varying distances, the bacterial suspension’s diffusion coefficient was determined to be 1.0 × 10^-8^–3.0 × 10^−8^ m^2^/s, which is higher than that of surfactants. Based on these findings, the optimal shut-in period was suggested to be 30 days. Microbial flooding experiments using the visual reservoir chip indicated that after 30 days of cultivation, the secondary water flooding recovery rate increased by 21.2% (from 28.4% to 49.6%). Emulsification and viscosity reduction by bacterial products, as well as the plugging of high-permeability channels by bacterial clusters, were likely key factors contributing to the improved recovery. This study quantitatively investigates bacterial growth dynamics at the pore scale using *in situ* observation techniques. The findings provide a theoretical foundation for microbial oil recovery and are significant for advancing its large-scale application.

## 1 Introduction

Microbial enhanced oil recovery (MEOR) is an emerging petroleum extraction technique that regulates reservoir systems by injecting selected exogenous microorganisms (Ke et al., 2019) or by stimulating the activity of indigenous microbial communities (Li et al., 2011). The metabolic products of microorganisms in reservoir environments, such as biosurfactants, organic acids, and gases, interact with the oil–rock–water interface through physicochemical reactions. These interactions can effectively reduce crude oil viscosity, lower interfacial tension, and modify rock wettability, ultimately improving oil recovery (Le et al., 2015; Niu et al., 2020). MEOR, by using microbial metabolites instead of conventional chemical agents, can significantly reduce environmental pollution during oil production. Its green and sustainable features are mainly reflected in three aspects: (1) Green feedstocks: Microorganisms use heavy fractions of crude oil or renewable carbon sources such as molasses, starch, and glucose as substrates (Liu et al., 2014). (2) Low-carbon processes: Metabolic products such as biosurfactants (e.g., rhamnolipids) and biopolymers (e.g., xanthan gum) can be degraded *in situ*, preventing reservoir contamination. Additionally, microbial-induced carbonate precipitation not only enhances oil displacement efficiency but also facilitates CO_2_ fixation during bacterial growth, thereby achieving the goal of “increasing oil recovery without increasing carbon emissions” (Lin et al., 2021). (3) Waste valorization: Oil recovery-associated microorganisms have the potential to bioremediate petroleum-contaminated environments (Deng et al., 2020) and can suppress the generation of toxic gases such as hydrogen sulfide (Ma et al., 2023), thereby promoting the synergy of oilfield development and ecological restoration. Therefore, MEOR, as a green oil recovery technology, has been widely used to improve tertiary oil recovery (Alkan et al., 2019; Zamani et al., 2020). However, the understanding of the growth characteristics and dynamics of microorganisms in reservoirs remains limited, which has slowed the early development of this technology in tertiary oil recovery (Wang, 2021).

In recent years, research on microbial oil recovery has expanded to various areas, including microbial modification of reservoir properties, microbial physiology and diversity, gene editing, and nanotechnology (Das et al., 2025; Elakkiya et al., 2020; Gao et al., 2020; Li et al., 2022; Ran, 2012). Many factors influence the efficiency of microbial oil recovery. These include reservoir conditions affecting microbial growth, such as temperature, pH, formation water salinity, and nutrient supply (Biktasheva et al., 2025), as well as factors influencing microbial transport and diffusion, such as reservoir permeability and porosity (Sen, 2008). Current studies on the mechanisms of microbial oil recovery are primarily based on physical simulations derived from past experience and oil displacement performance (Ke et al., 2018). However, experimental limitations often lead to significant discrepancies between laboratory results and field application data.

Microfluidic technology, derived from the liquid-handling component of microelectromechanical systems (MEMS), is designed to process liquids at the micro- or nanoscale (Fan et al., 2018). In oil recovery studies, microfluidic technology is primarily used to replicate microscopic porous media structures on microfluidic chips, which are then used in physical simulation experiments to analyze displacement mechanisms (Wang et al., 2025). This approach effectively simulates how various displacement agents impact oil recovery under rock pore conditions. Benefiting from advances in visualization and microfluidic technologies, combined with CT core scanning, it is now possible to more directly observe microscopic oil displacement processes and elucidate the underlying mechanisms (Wu et al., 2024). Today, the integration of visualization and microfluidic technologies is widely used to study multiple displacement methods, including CO_2_ flooding, surfactant flooding, polymer flooding, foam flooding, and microbial flooding (Guo and Aryana, 2016; Li et al., 2025; Liu et al., 2023; Xu et al., 2020).

The integration of visualization and microfluidic technologies has made significant progress in the field of microbial oil recovery. Park et al. (2023) examined how the transition from oil-wet to water-wet conditions during bacterial growth and biosurfactant production influences immiscible fluid flow at the pore scale. Shabani et al. (2024) used visual micromodels to evaluate the performance of biosurfactants produced by *Enterobacter cloacae* and *Acinetobacter* calcoaceticus, systematically analyzing the effects of salinity, oil displacement method, and bacterial species on improving oil recovery. Karambeigi et al. (2015) employed micro-visual models to study the impact of microbial plugging on water injection efficiency, finding that permeability differences significantly affected water flooding, with high-permeability zones performing much better than low-permeability zones. Wu et al. (2024) developed a middle-phase microemulsion flooding system and, through phase change and microscopic residual oil distribution experiments, revealed its micro-displacement mechanisms. However, further studies are still needed on *in-situ* microbial observation and microscopic mechanisms.

This study utilizes microfluidic chip technology to construct a visual microfluidic model at the pore scale of reservoirs. By simulating realistic reservoir pore structures and integrating microscopic *in-situ* observation techniques, the research examines microbial growth patterns, diffusion characteristics, and oil displacement mechanisms within pores. The findings are expected to enrich the fundamental theoretical framework of microbial oil recovery and provide valuable data support for future studies in this area.

## 2 Experimental procedures

### 2.1 Materials

In this study, all oil and water samples were prepared in the laboratory. The mineral oil was formulated by blending oils of 500 mPa·s and 120 mPa·s viscosity to obtain a target viscosity of 300 mPa·s. Viscosity was measured with an NDJ-5S viscometer. The oil was then stained with 1.2% Oil Red O (C_26_H_24_N_4_O), filtered, and stored for use. The displacement water was deionized water with added dyes. For primary water flooding, 1.5% methylene blue solution was used and filtered for use. For secondary water flooding, green food coloring (7 drops per 20 mL) was used as the dye. The WJ-8 bacterial suspension used in the experiments was provided by the Shengli Oilfield Research Institute. It belongs to the *Pseudomonas* genus and can synthesize rhamnolipids (Cao et al., 2022; Chen et al., 2023) through the glycolipid pathway. The nutrient medium, also supplied by the institute, contained the necessary carbon and nitrogen sources for bacterial growth. The bacterial suspension was refreshed every 3 months to maintain activity. Before use, it was stored at 4 °C, shaken to homogenize, and then the required volume was extracted using a sterile syringe. The suspension was mixed directly with nutrient medium, shaken thoroughly, and prepared for immediate use.

### 2.2 Microbial visualization reservoir chip manufacturing

**FIGURE 1:**
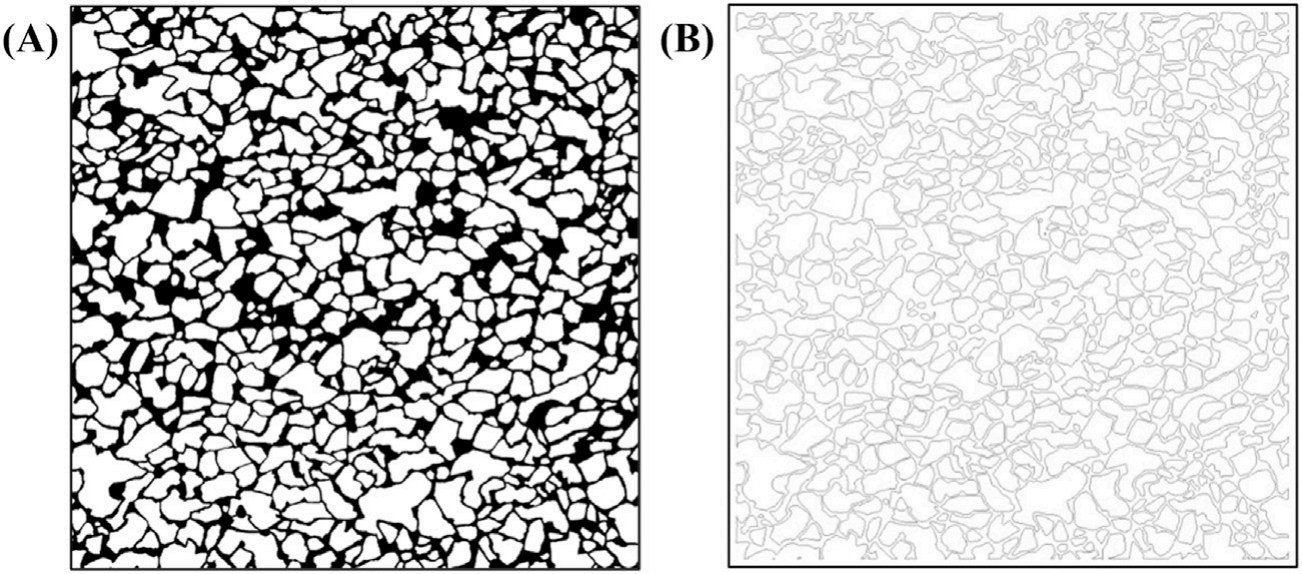
Contains subfigures A and B. Subfigure (**A**) shows the pre-processed digital chip with densely distributed irregular black-and-white patterns and distinct black borders. Subfigure (**B**) shows the post-processed geometric structures for laser lithography, with lighter tone, lower contrast, and more pronounced contours.

This study utilized digital rock modeling and microfluidic fabrication techniques to design and produce two types of visual reservoir chips tailored to the research objectives: a microbial flooding chip and a microbial observation arched chip (each designed for specific purposes). The detailed fabrication process is as follows:

First, core samples from the Shengli Oilfield were scanned using an X-ray micro-computed tomography system (Xradia 630 Versa, United States) to construct a high-resolution three-dimensional digital rock model. Morphological erosion and dilation operations were repeatedly applied to the segmented images, which had been median-filtered, based on 2D core slices and pore network numerical reconstructions, in order to further suppress noise. To address the field-of-view limitations of CT scanning, multiple scans and binary processing were carried out, followed by image stitching to generate large-scale core images measuring 120 mm × 120 mm (Figure 1) (Zhang et al., 2022). Subsequently, laser lithography without a mask was applied using an infinite stitching approach. The laser frequency was set to 60 kHz, and four exposures were performed, achieving a final depth of approximately 150 μm, in accordance with the 2D pore-scale digital core design for etching the glass substrate. No additional surface treatment was applied to the glass beyond the lithography process.

After completing the fabrication of microchannels and injection holes by laser lithography, ultra-clear soda-lime glass plates of the same size and material were used as cover plates. The laser-ablated substrates containing the microchannels and porous structures were ultrasonically cleaned sequentially in the following solutions: ① 2 g/L sodium silicate–acetone mixture, ② 2 g/L surfactant solution, and ③ deionized water After 30 min of ultrasonic cleaning, the substrates were air-dried in a cleanroom, stacked with the cover plates, and then sintered in an external-heating vacuum furnace at 650 °C. The reservoir chips were made of ultra-clear soda-lime glass, with dimensions of 150 mm × 150 mm and a thickness of 6 mm.

**FIGURE 2:**
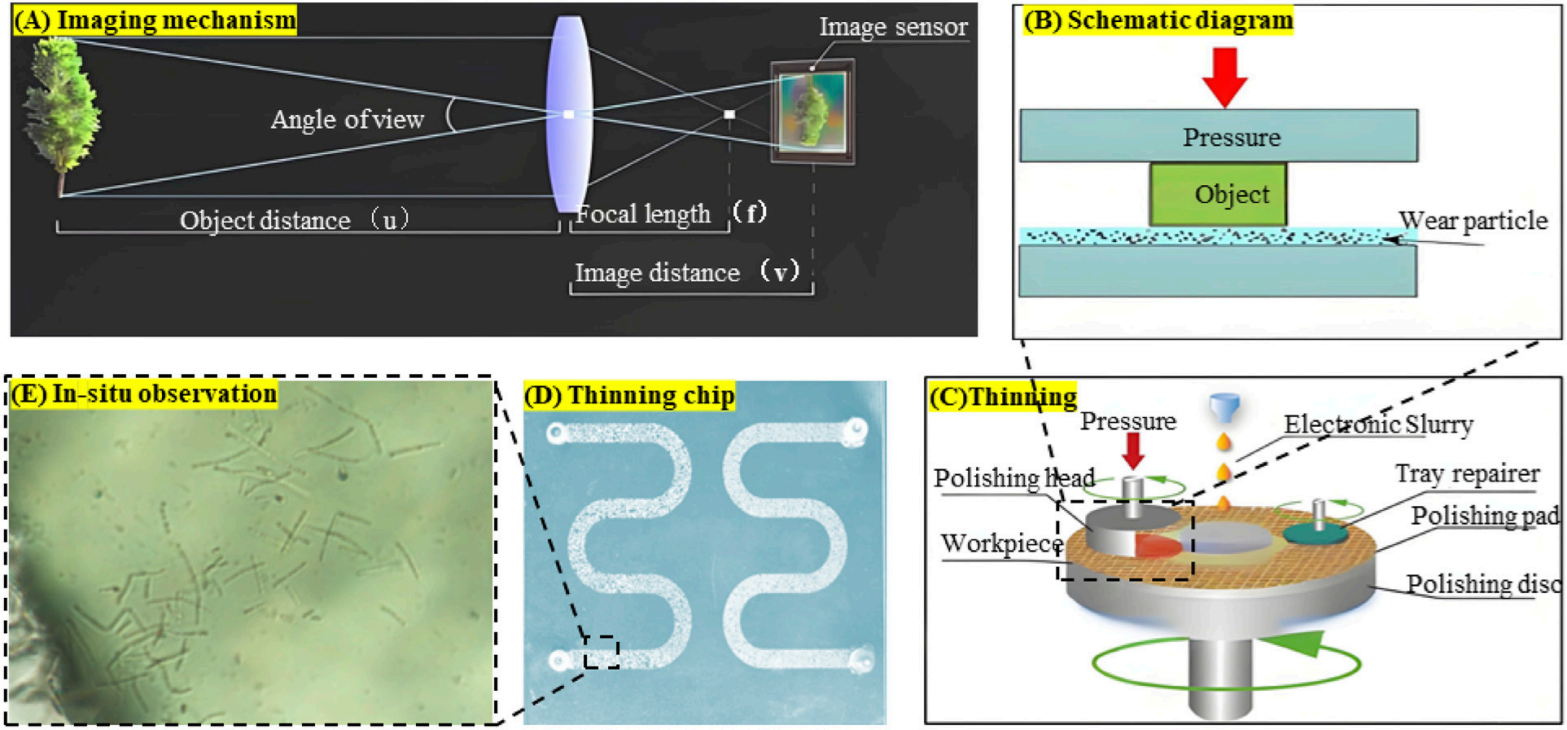
Visual chip production process: **(A)** Imaging principle of the chip under the microscope, **(B)** Grinding principle, **(C)** Chip thinning process, **(D)** Chip after thinning, **(E)** *In-situ* observation results.

For the arched chips used in microbial observation experiments, additional processing steps were required beyond those described above. A grinding machine was used to thin and polish the underside of the chip to achieve clearer imaging of microorganisms, thereby improving the quality of microbial growth observations. In summary, the fired chip was mounted on a polishing head, ground with 10 μm slurry and a 300-mesh plate under 30 kg pressure for 15 min per cycle (removing ∼0.5 mm per cycle), and then polished with a cerium oxide plate and 200 nm slurry under identical conditions until the surface was transparent. Repeated experiments determined that thinning the chip to 0.2 mm provided the optimal thickness, balancing both imaging quality and mechanical strength. The detailed processing procedure and principle are shown in Figure 2.

### 2.3 Visual microfluidic device

#### 2.3.1 Microbial growth observation

**FIGURE 3:**
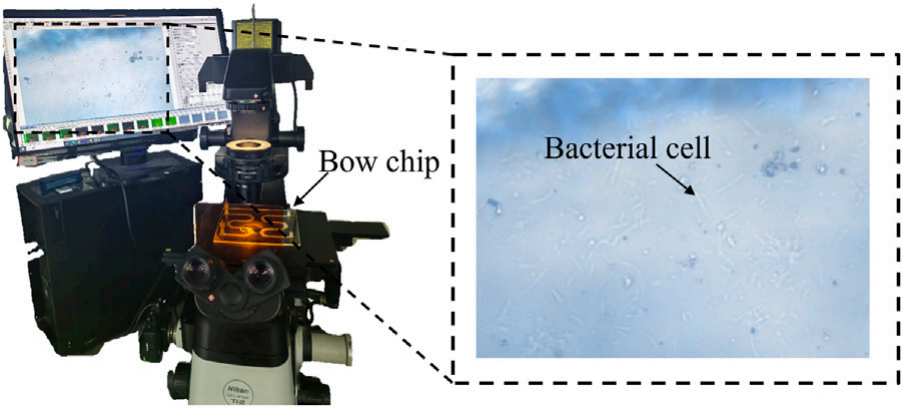
*In-situ* observation equipment.

After the chip thinning process was completed, bacterial growth dynamics were observed using a Nikon ECLIPSE-Ti2 microscope (Figure 3), with images and videos captured through the NIS-Elements D software. This microscope system was primarily used to study bacterial growth curves, diffusion coefficients, and overall growth patterns. Observations were conducted by monitoring fixed regions at magnifications of 200× and 500×, with videos recorded simultaneously to quantify bacterial populations.

#### 2.3.2 Microbial flooding observation

**FIGURE 4:**
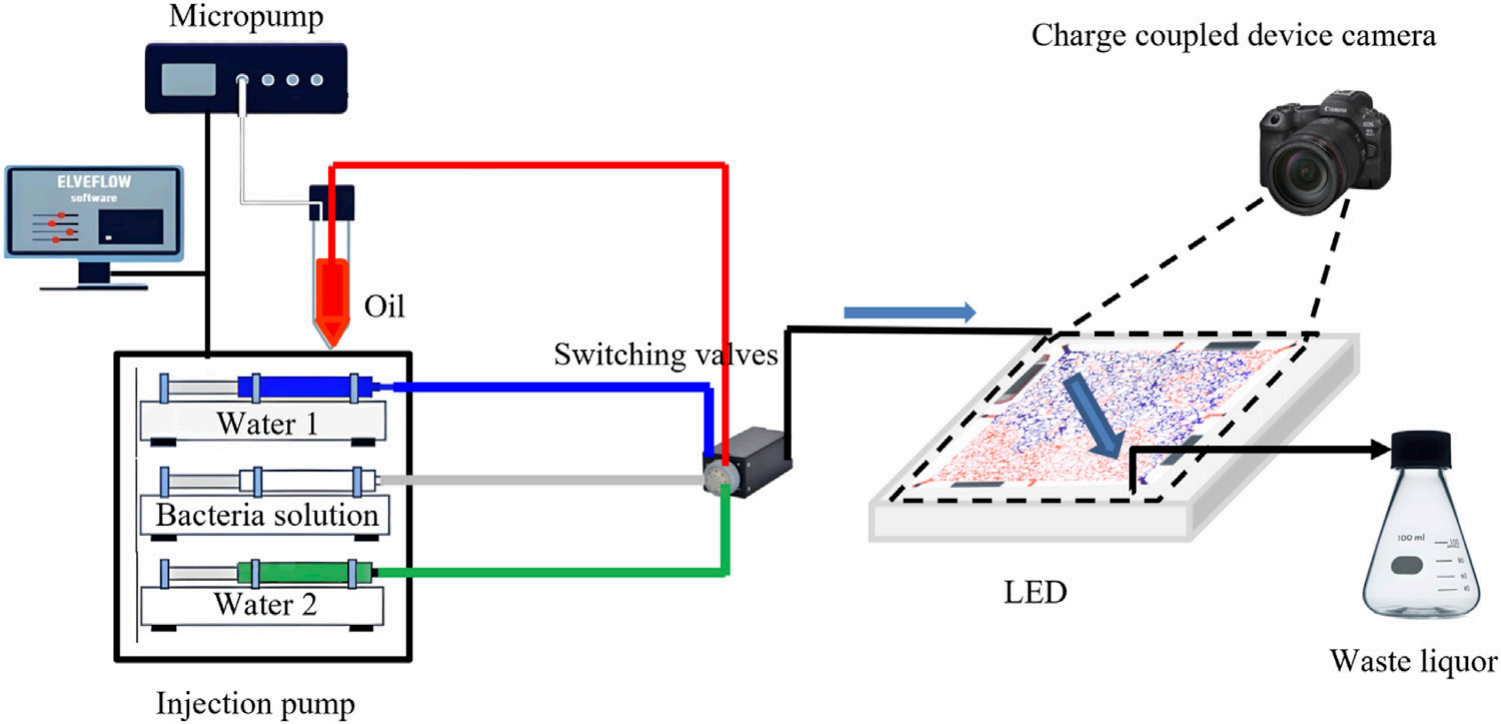
Visual microfluidic oil displacement experimental process.

The visual microfluidic setup used for microbial oil displacement experiments is shown in Figure 4 and consists of the following components: (1) An Elveflow microfluidic pump (France) set to maintain a saturated oil pressure of 1000 mbar; (2) A high-precision Cetoni syringe pump (Germany) that ensures a stable displacement rate for both water flooding and microbial flooding; (3) A charge-coupled device (CCD) camera mounted on a tripod directly above the microfluidic chip for continuous image capture. The image acquisition interval, determined by the displacement rate, ranged from 5 to 20 s; (4) An LED light array to provide stable illumination; (5) A waste collection conical flask for storing discharged fluids.

### 2.4 *In-situ* observation of microbial experimental process

#### 2.4.1 Microbial growth observation

**FIGURE 5:**
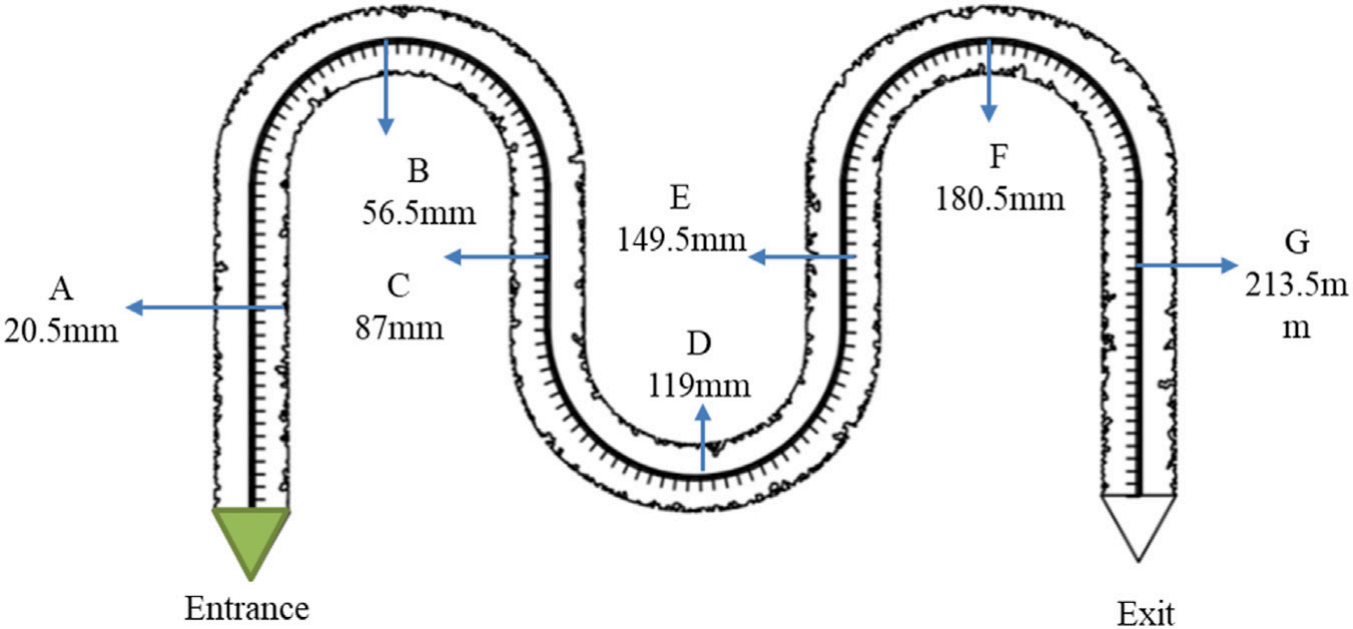
The experimental point setting of growth law.

In this study, bacteria were injected into low-permeability channels to establish a multi-point monitoring system (Figure 5). Over a 5-day observation period, the system was used to investigate the dynamic characteristics of bacterial growth. Seven fixed monitoring points were established in the experiment to comprehensively analyze changes in bacterial population size, morphological evolution, and colony diffusion. The detailed procedure was as follows:

Observation points were evenly distributed along the arched chip channels, named A through G from the inlet to the outlet. The distance between adjacent points was approximately 30 mm.

The channels were first rinsed thoroughly with anhydrous ethanol and dried using nitrogen gas. A microfluidic pump was then used to saturate the channels with a standardized nutrient solution to provide sufficient carbon and nitrogen sources for the bacteria. Next, a microfluidic pump was used to inject WJ-8 bacterial suspension stained with green dye until the outlet turned completely green. Images were captured at the fixed observation points at scheduled intervals. The observation period was set at 5 days, with daily microscopic monitoring to record bacterial population, morphological changes, and colony diffusion.

Bacterial growth was quantified using growth curves modeled by the Logistic growth model. This model describes population growth under specific environmental conditions by incorporating the concept of carrying capacity (maximum population size). Initially, growth is rapid, but as the population approaches the environmental carrying capacity, the growth rate slows and eventually stabilizes.

The cumulative function used to describe bacterial growth in the model is as follows:

$$y=k/\left(1+ae^{-bt}\right)$$ (1)

Equation 1 represents the cumulative function of bacterial growth. Here, y denotes the bacterial count, and k is the bacterial growth rate b denotes different resource concentrations, and a is a differential constant. When the growth time t is sufficiently large, the value of y approaches that of k (Wilson, 1994).

$$t_{1}=\frac{lna-1.317}{b}$$ (2)

$$t_{2}=\frac{lna}{b}$$ (3)

$$t_{3}=\frac{lna+1.317}{b}$$ (4)

In Equation 2, t1 marks the boundary between the lag phase and the early growth phase. Equation 3 can be used to calculate t_2_, the time point at which the growth rate reaches its maximum, representing the boundary between the early and late growth phases. t_3_ denotes the boundary between the late growth phase and the stationary phase.

#### 2.4.2 Determination of microbial diffusion coefficient

The experiment was designed to monitor the furthest distance that bacteria spread within the channels at fixed time intervals after injection, in order to determine the bacterial diffusion coefficient in the channels.

It should be noted that in previous experiments, arched chips were used to study bacterial growth. Although these chips offered certain experimental advantages, they also had limitations. Multiple blind-end regions within the chips were difficult to clean thoroughly due to their design. Even after repeated rinsing with anhydrous ethanol, trace amounts of bacteria could remain in these areas, potentially affecting bacterial counts and overall experimental accuracy.

**FIGURE 6:**
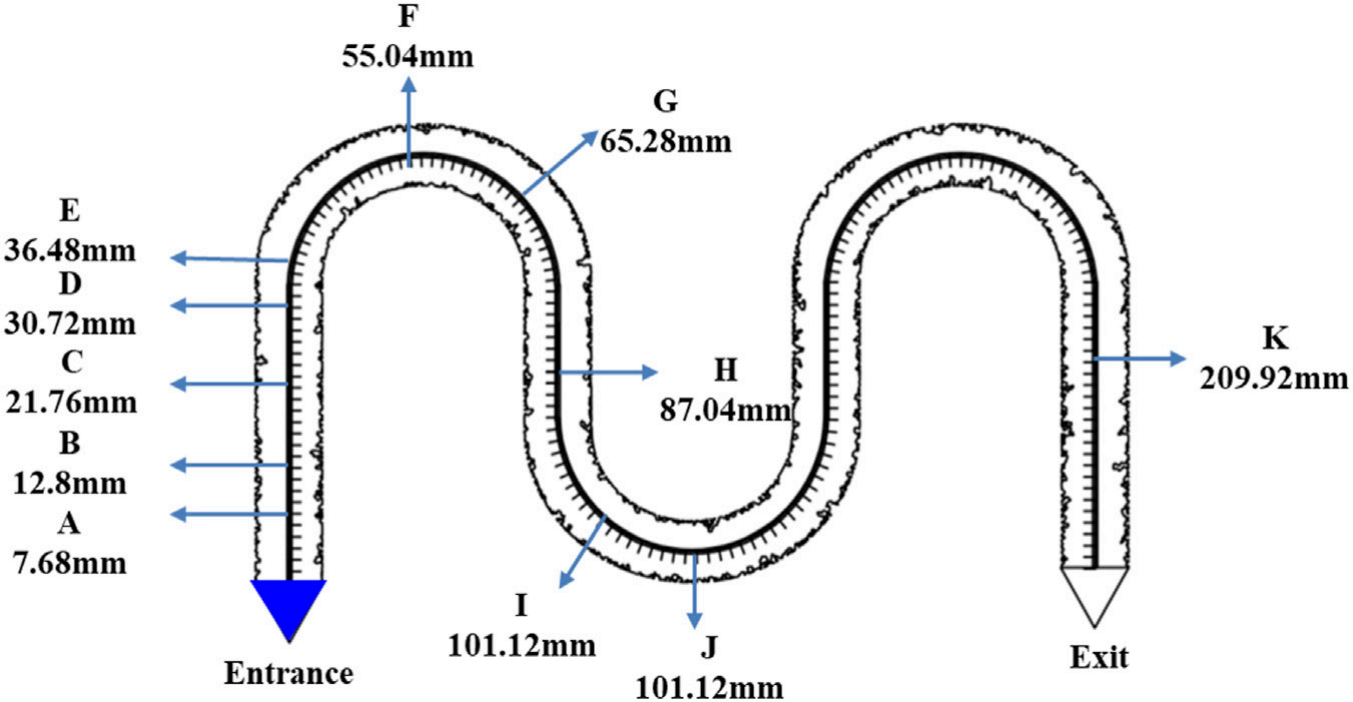
Experimental point setting of permeability coefficient.

To more accurately evaluate bacterial growth and minimize experimental errors, a correction method was designed. As shown in Figure 6, the downstream location near the channel outlet was selected as the background reference point (denoted as point K). Because of its distance from the injection site, bacterial diffusion at this point was minimal, making it a stable reference region unaffected by bacterial growth. During each measurement, bacterial counts at Point K were recorded as background values. For bacterial counts at other observation points, the background count was subtracted from the observed counts. This process reduced systematic errors caused by residual bacteria and incomplete cleaning in arched chips, thereby improving the accuracy and reliability of the experimental data.

The detailed experimental procedure was as follows: First, an 80% WJ-8 bacterial suspension was prepared by mixing the suspension with nutrient medium at a 4:1 volume ratio. The WJ-8 suspension was then stained to facilitate clear microscopic observation of bacterial diffusion. Green food dye (7 drops per 20 mL) was used for staining, consistent with the dye and concentration applied in the secondary water flooding stage. After staining, the bacterial suspension was injected into the pore channels using a syringe pump until the inlet triangular region was completely filled.

After bacterial injection, the diffusion process was observed microscopically at fixed time intervals. Observation points were arranged based on diffusion principles: closer spacing near the inlet and wider spacing at the downstream end. The observation interval was set at 2–3 h, with adjustments made depending on the actual diffusion behavior of the microorganisms.

The diffusion distance at each time point and the bacterial concentration were recorded and used in subsequent calculations. Based on bacterial activation time and distance, the diffusion coefficient of the nutrient solution in water was derived using the following formula. Specifically, when C/C_0_ reached 50%, this concentration was defined as the effective concentration. The activation concentration was determined using a cell-counting algorithm. By tracking changes in cell numbers over time, a fitted growth curve was generated to identify the specific time point at which the bacterial concentration reached C/C_0_ = 50%. By combining time and distance variables, the range of diffusion coefficients could be determined.

The formula used to determine the diffusion concentration of the bacterial suspension is as follows:

$$c=c_{0}erfc\left(\zeta \right)$$ (5)

$$\zeta =\left(z/{\left(4Dt\right)}^{1/2}\right)$$ (6)

In the above formula, *c* represents the concentration at a position *z* from the injection point at time *t*; *c*_*0*_ is the initial concentration (at the injection point); *erfc* denotes the complementary error function; *z* is the distance from the injection point (m); *D* is the diffusion coefficient (m^2^/s); and *t* is the time (s).

#### 2.4.3 Physical simulation experiment of microbial enhanced oil recovery

The microbial oil displacement experiments were conducted using 150 mm × 150 mm chips for physical simulation, and the detailed procedure is as follows: The diagonal direction of the chip was selected as the main fluid channel. First, the chip was saturated with oil (viscosity 300 mPa·s) using an Elveflow microfluidic pump. Primary water flooding was then performed using a Cetoni high-precision syringe pump, injecting deionized water stained with methylene blue at a rate of 15 μL/min. The primary water flooding ended when the flooded area no longer expanded.

After water flooding, the bacterial suspension was injected using the syringe pump at a concentration of 20% and a flow rate of 15 μL/min. Injection was stopped once the bacterial suspension fully penetrated the channels and the displacement area ceased to change.

Next, a microbial huff-and-puff experiment was conducted by placing the chip in a constant-temperature incubator at 60 °C for a shut-in period of 30 days. After the shut-in period, secondary water flooding was performed by injecting green-stained deionized water using the syringe pump until the flooded area no longer expanded.

Finally, images were processed using MATLAB software for color recognition to calculate the recovery factor at each stage. The cumulative changes in recovery were analyzed to verify the appropriateness of the experimental parameters.

The specific parameters and procedures for image processing and color recognition using MATLAB are as follows: In this study, image segmentation and target recognition were performed in the HSV (hue–saturation–value) color space rather than RGB. The HSV space effectively separates color information from luminance, reducing the influence of illumination fluctuations on recognition results. This approach is more suitable for distinguishing oil, water, and stained bacteria within reservoir pore structures.

The image processing workflow and parameter settings are as follows:

The original RGB microscopy images were converted to HSV format using built-in MATLAB functions, and the hue (H), saturation (S), and value (V) channels were extracted as matrices.

Based on the color characteristics of different targets (oil, water, and stained bacteria), HSV threshold ranges were determined through preliminary experiments to construct binary masks for region extraction. The thresholds are defined as:

Oil phase (red): Stained with Oil Red, hue concentrated at high and low values:

redMask = (H > 0.85 |H < 0.05) & S > 0.2 & V > 0.3;

Water phase (blue): Stained with methylene blue, hue between 0.55 and 0.75:

blueMask = (H > 0.55 & H < 0.75) & S > 0.2 & V > 0.3;

Secondary water phase (green): Stained bright green, hue between 0.2 and 0.4, with increased value threshold to separate from background:

greenMask = (H > 0.2 & H < 0.4) & S > 0.2 & V > 0.5;

Saturation and value constraints were applied to suppress background noise and low-saturation interference.

## 3 Results and discussion

### 3.1 Microbial growth characteristics

#### 3.1.1 Microbial growth curve

In this study, an *in-situ* observation method was employed by selecting seven representative points within the channels for a 5-day observation period, ensuring that the collected data reflected the growth patterns within the pore system. These points encompassed all channel structures, including blind ends, throats, and main channels. Because bacteria not only form stable colonies during growth but also exhibit motility and diffusion, observations at the same location may show minor fluctuations. Therefore, outlier data were removed, and growth curves were fitted using multiple datasets to improve result reliability.

Anomalies were defined as sudden unexpected changes in cell counts. For example, on day 4, point D showed 105 cells in the field of view, but on day 5, due to increasing bubbles, only 37 cells were observable. Similarly, during rapid growth, any abrupt decrease caused by cell movement is considered an outlier and removed.

Based on these considerations, three observation points—D, E, and F—were ultimately selected, all located in the latter half of the transport route. The likely reason is that after the bacterial suspension entered the throat water body, a concentration gradient was formed due to density differences. Bacteria diffused from high-to low-concentration areas, resulting in more significant fluctuations in bacterial counts in the upstream region. In contrast, the downstream region exhibited a more gradual concentration gradient, with fewer fluctuations and more stable bacterial growth.

**FIGURE 7:**
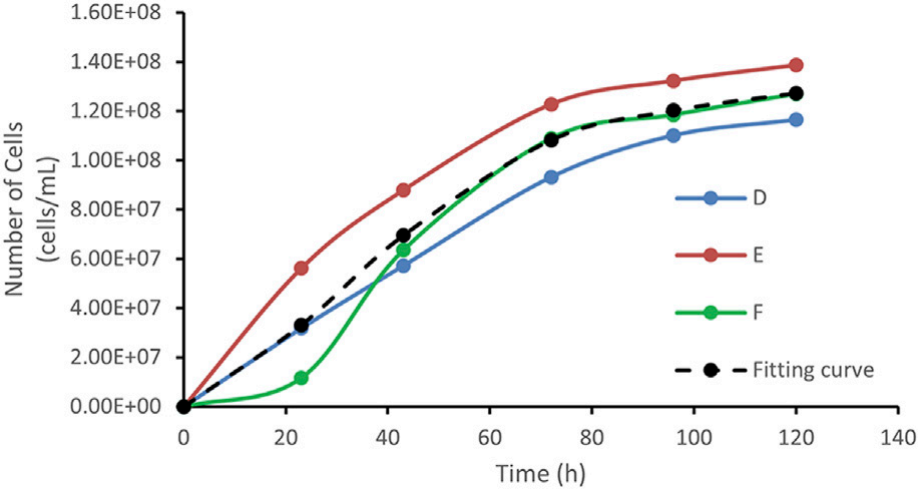
Growth curves of D, E, F points, and fitting curve.

Figure 7 shows the bacterial count curves for points D, E, and F. The bacterial population trend at point F closely matched the standard growth curve, with clearly distinguishable lag, exponential, and stationary phases. By contrast, points D and E only exhibited the exponential and stationary phases, without displaying the full bacterial growth cycle.

**FIGURE 8:**
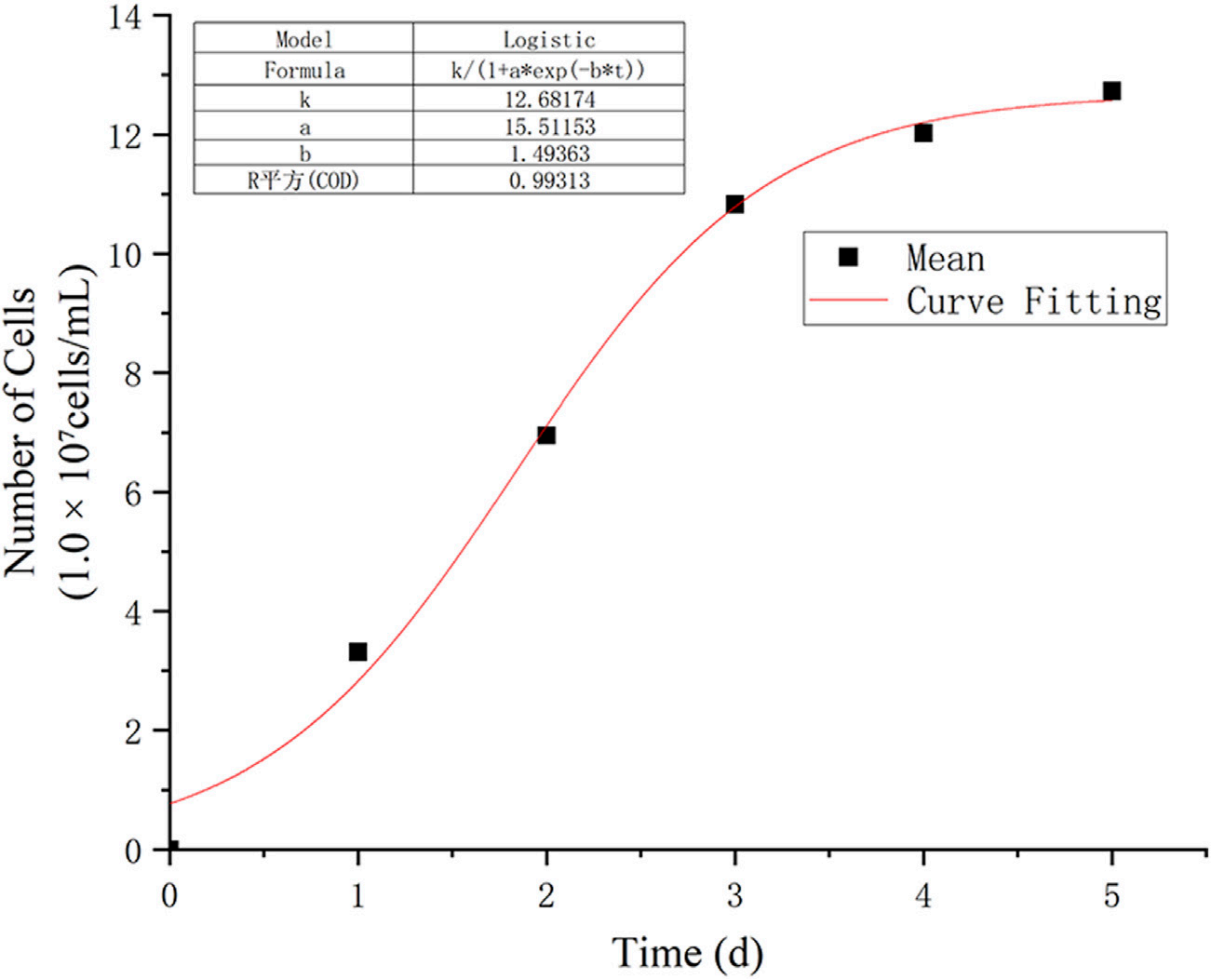
Logistic fitting growth curve.

In this study, the observation data from points D, E, and F were averaged and subjected to nonlinear curve fitting using Origin software. The Logistic function under the Growth/Sigmoidal category was selected for the fitting. The fitting results are shown in Figure 8, and the corresponding parameters are listed in Table 1:

**TABLE 1:** Growth curve parameters.

Parameter	Value	Standard error
k	12.6817	0.4821
a	15.5115	5.7729
b	1.4936	0.2187

The R^2^ of the fitting curve reached 0.993, indicating a high goodness of fit. Based on the results calculated from Equation 4, when these findings are extrapolated to real oilfield production, the shut-in incubation stage for bacteria should allow at least 2.71 day after bacterial diffusion and stabilization in the waterflood channels. This ensures the bacteria enter the stationary phase and begin secreting biosurfactants or viscous substances.

It is important to note that because the experimental conditions were relatively ideal, the time required for bacteria to adapt to the actual reservoir environment may be longer. Various factors such as formation water salinity, pH, and temperature could extend the lag phase beyond the 22.89 h indicated by the fitted *t*_*1*_ value.

**FIGURE 9:**
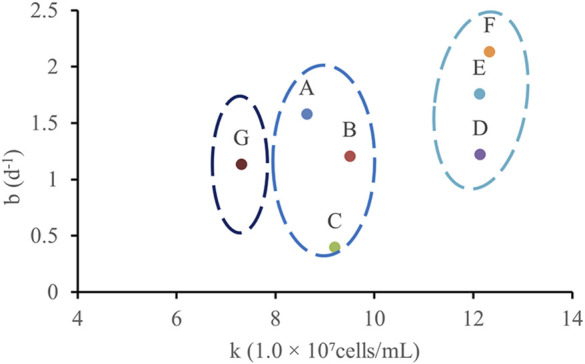
The corresponding relationship between k and b at different points is obtained by fitting.

According to the cumulative growth function (Equation 1): By applying the same curve fitting method to the data from points A to G, the correlation between the parameters *k* and *b* was determined, as shown in Figure 9.

The analysis led to the following conclusions: (1) The rapid growth phase of WJ-8 bacteria lasted 1–3 days, after which they entered the stationary phase. This finding can provide a basis for determining microbial flooding parameters; (2) Under the concentration conditions at points D, E, and F, WJ-8 bacteria exhibited faster growth, moderate growth at points A, B, and C, and the slowest growth at point G; (3) The arched chip structure showed slower growth at both ends and faster growth in the central region.

The term “microbial huff-and-puff experiment” refers to maintaining microbial culture conditions (e.g., temperature control, nutrient supply) in a closed system to promote biological reactions. When the growth curve transitions from the logarithmic (exponential) phase to the stationary phase, microorganisms begin secreting metabolic products and forming biofilms. This process is associated with nutrient depletion and environmental stress factors. The critical turning point occurred on day 3, indicating that days 2–3 represent the optimal incubation window. Maintaining favorable conditions during this period can maximize microbial activity and product accumulation efficiency.

#### 3.1.2 Analysis of microbial growth characteristics

**FIGURE 10:**
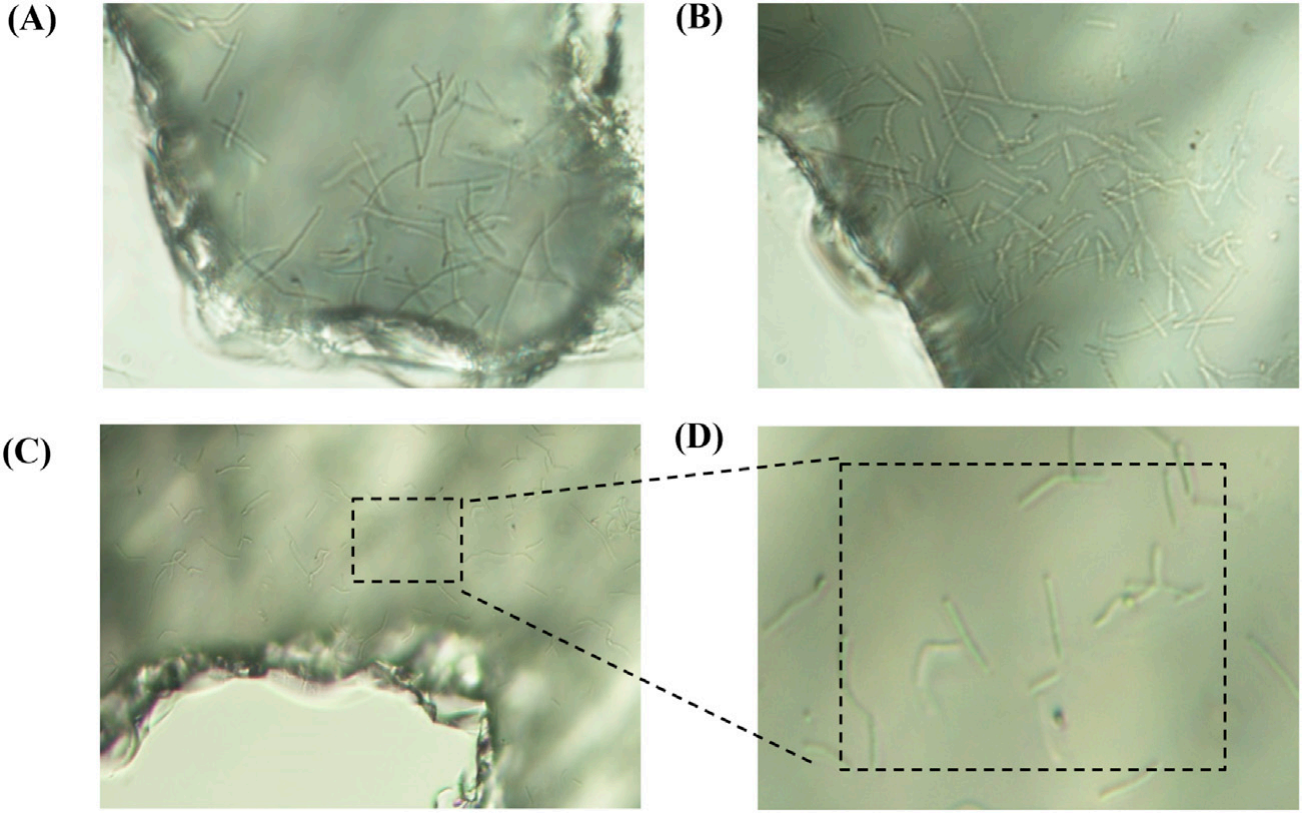
The growth characteristics of microorganisms in the pores, **(A)** represents the growth in the blind end, and **(B)** represents the growth of the roar edge, **(C,D)** are used to observe the growth inside the roar.

Figure 10 The growth characteristics of microorganisms in the pores, A represents the growth in the blind end, and B represents the growth of the roar edge, C and D are used to observe the growth inside the roar.

##### 3.1.2.1 Bacteria form clusters at blind ends of throats.

At point B, located in the blind-end region of the throat, significant bacterial aggregation was observed (Figure 10A). Blind ends represent terminal areas of water or oil/gas flow paths, characterized by poor flow and abundant attachment sites, making them hotspots for bacterial aggregation. There are two main reasons why bacteria begin to proliferate here when flow velocity decreases or stagnates: (i) in low-flow regions, the shear force is reduced, making it easier for bacteria to attach to solid surfaces or oil droplets; and (ii) blind ends provide numerous attachment sites, such as mineral surfaces or oil droplets, further promoting bacterial clustering.

Bacterial aggregation is a manifestation of self-organization, leading to cluster formation. This clustering enhances biofilm development, increasing bacterial adhesion and survivability. In poorly flowing blind-end regions, bacterial clusters can resist water flooding and fluid impact. Additionally, secreted enzymes or biosurfactants can break down oil droplets or alter oil properties, thereby improving oil displacement efficiency in these regions.

At blind ends, the low water flow velocity and poor mobility often hinder effective oil displacement further downstream. The tendency of bacteria to aggregate in these areas suggests that they may possess greater potential for enhanced oil recovery, particularly in hard-to-produce reservoirs. Bacterial clustering may not only improve water flooding performance in blind ends but also enhance residual oil displacement efficiency, offering a promising reservoir modification strategy.

##### 3.1.2.2 Bacteria grow more diffusely in the throat interior.

Under 200× magnification, the throat interior at point F (Figures 10C,D) exhibited a distinctly dispersed bacterial growth pattern. Unlike the clustered colonies in blind-end regions, bacterial growth here was uniformly distributed, making high-density clusters difficult to form.

This phenomenon may be related to the following factors:

1. Flow heterogeneity: Compared to the edges, the interior of the throat likely experiences more uniform flow and stable velocities, resulting in evenly distributed bacteria and fewer dense clusters;
2. Limited attachment sites: The throat interior offers fewer attachment surfaces, reducing opportunities for bacteria to adhere and aggregate;
3. Nutritional and environmental conditions: Differences in hydrodynamic and chemical environments may affect bacterial growth patterns, especially when suitable surfaces or microenvironments for aggregation are lacking.

##### 3.1.2.3 Bacteria readily form large clusters at throat edges.

Analysis of multiple observation points revealed that bacteria tend to form large clusters at the edges of throats (Figure 10B). This phenomenon may be closely related to the distribution of attachment sites and flow velocity differences.

1. Boundary effects: Throat edges typically feature boundary layers with slower flow velocities and weaker turbulence, creating favorable conditions for bacterial growth, adhesion, and aggregation. In contrast, the faster flow in the throat center generates higher shear forces that disperse bacteria and inhibit cluster formation. A similar phenomenon occurs in the human upper respiratory tract, where low-speed airflow regions provide ecological niches for bacteria (Odendaal et al., 2024).
2. Abundant attachment sites: Throat edges typically provide more physical attachment points (e.g., mineral surfaces, filling material), offering bacteria a habitat where they can settle, reproduce, and form clusters.

These observations suggest that bacterial community structures at throat edges may be more advantageous for enhancing oil recovery. The clustering of bacterial communities could provide stronger support for mobilizing otherwise hard-to-displace oil. Therefore, in microbial oil recovery applications, considering factors such as flow velocity and attachment site distribution to optimize bacterial community distribution can help improve reservoir development efficiency.

### 3.2 Microorganism diffusion law

In the bacterial growth observation experiment, bacterial counts at 11 observation points were recorded at different time intervals (Table 2). By subtracting background values and setting a baseline for bacterial activation, the relationship between activation points and activation times was determined. Using this method, activation data for the observation points were obtained: four points (A, B, C, and E) were activated at 3, 7.5, 10.5, and 14 h, respectively.

**TABLE 2:** The number of bacteria at each point during the observation time.

Time (h)	Position										
	A	B	C	D	E	F	G	H	I	J	K
2	16	8	10	7	15	3	8	4	7	8	6
4	22	7	8	6	14	4	9	4	9	8	8
6	30	8	5	8	16	5	10	6	9	7	9
9	29	40	9	9	20	6	8	9	11	8	10
12	31	55	20	10	23	7	8	11	15	9	13
15	42	76	26	20	56	13	6	16	16	6	8
17	37	41	28	12	58	17	10	21	12	13	11

**FIGURE 11:**
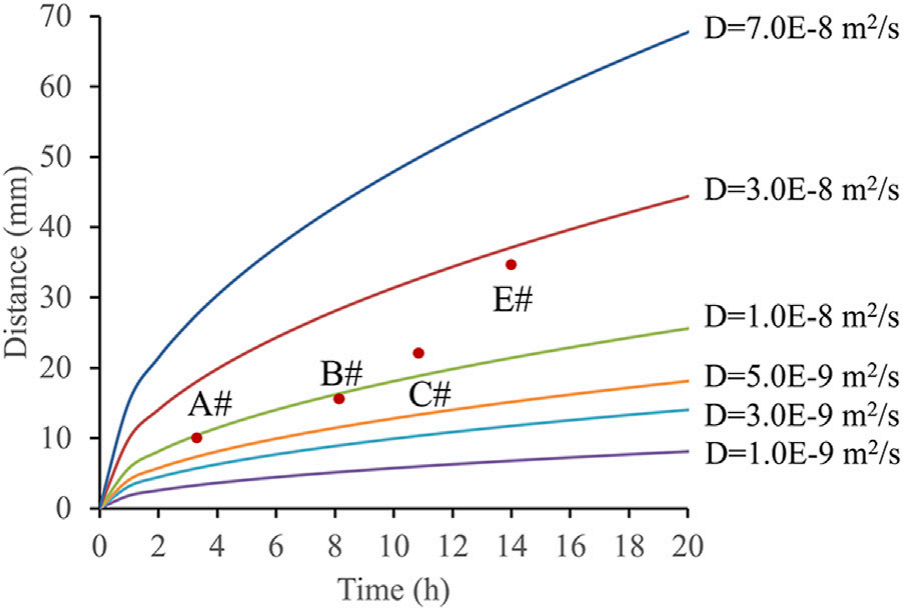
Microbial migration distance under different time and different diffusion coefficient conditions.

Based on the activation times and distances of the bacteria, the diffusion coefficient of the nutrient solution in water was calculated (Figure 11). The concentration at which C/C_0_ reaches 50% was defined as the effective concentration. By combining time and distance variables, the range of diffusion coefficients was determined. Using the known activation points and times, these locations were plotted on the charts. Measurement data from points A, B, C, and E were used to further derive the bacterial diffusion coefficient range, which was found to be 5.0 × 10^−9^ to 3.0 × 10^−8^ m^2^/s.

The experiment revealed that diffusion coefficients were lower near the injection port, but increased progressively with distance from the injection port. This phenomenon may be related to the bacterial activation process, the penetration characteristics of the nutrient solution in water, and changes in fluid dynamics. Near the injection port, steep concentration gradients and complex fluid flow conditions likely reduced the diffusion rate, whereas further away, less fluid disturbance allowed the diffusion coefficient to increase. The diffusion coefficient of the bacterial suspension was significantly higher than that of surfactants (1.0 × 10^−10^ m^2^/s) (Druetta and Picchioni, 2020).

### 3.3 Characteristics of actual microbial oil displacement

Experimental parameters obtained from the study of bacterial growth patterns and diffusion coefficients were used to determine the optimal shut-in time. Subsequently, microbial oil displacement experiments were conducted using large-scale reservoir chips (150 mm × 150 mm).

Determining the bacterial shut-in period required consideration of multiple factors affecting their growth cycle. Since bacteria typically need 5–7 days to reach the stationary phase and the use of methylene blue-stained water for water flooding may extend the lag phase, it was assumed that 10 days were required for bacteria to reach the stationary phase. Bacterial diffusion within the channels was also considered. The approximate distance for bacteria to diffuse from the main channel to the terminal branch was 10 mm. Using the lowest diffusion coefficient and Equations 5, 6, the time required for diffusion to the terminal branch of the waterflood side channel was calculated as 8.14 days. In addition, bacteria begin producing biosurfactants after entering the stationary phase, which requires at least 24 h and typically 3–7 days. Considering that the recommended shut-in period for medium-to low-viscosity crude oil (200–1000 mPa·s) is 7–30 days, suitable for conventional heavy oil, bacterial metabolites (e.g., biosurfactants, organic acids) can quickly improve crude oil mobility (Shi et al., 2023; Yin et al., 2023).

**FIGURE 12:**
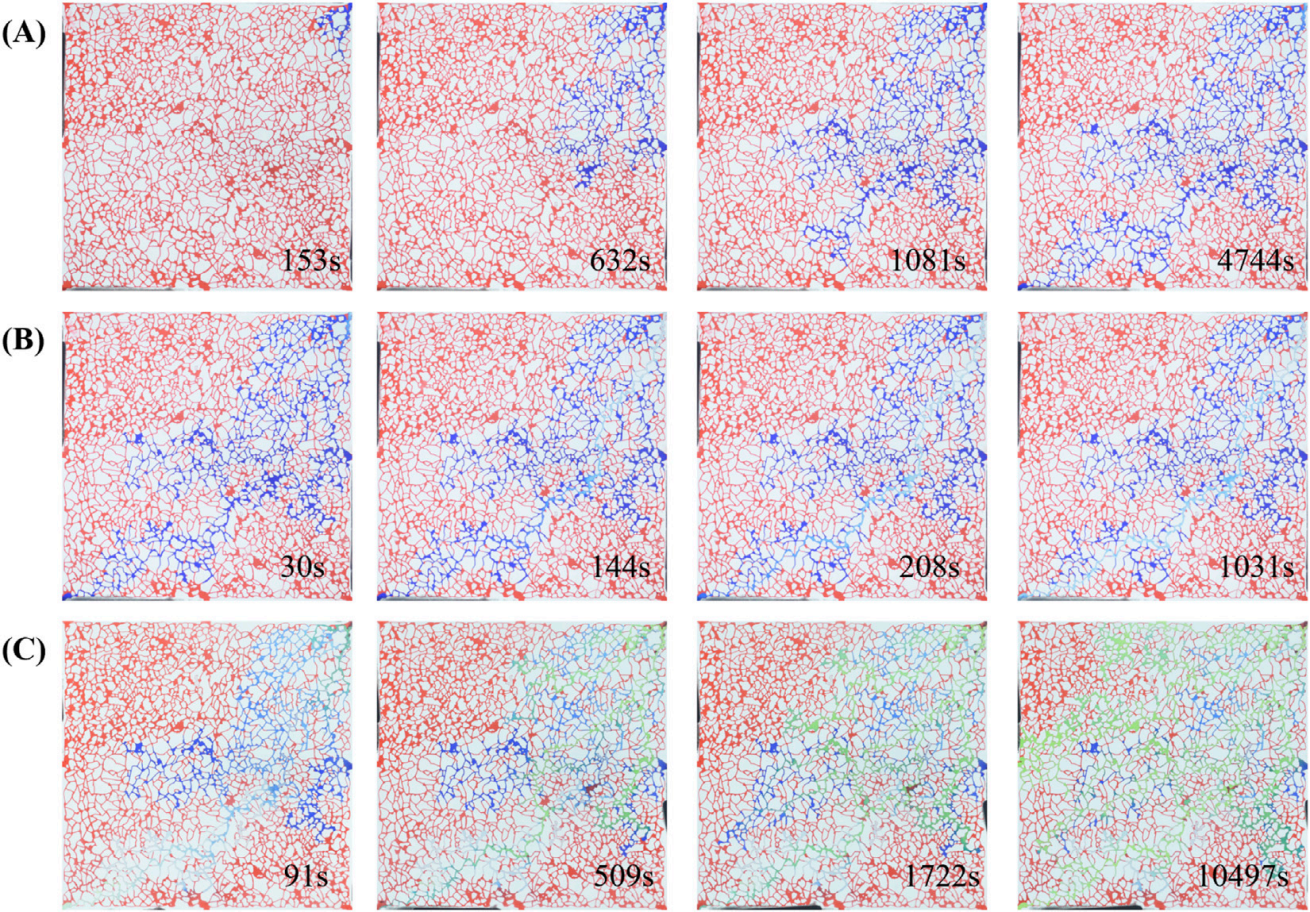
Three sets of time-lapse images (**A‐C**) showing network structure changes over time. Each set contains 4 images corresponding to different time points: Set A (153s, 632s, 1081s, 4744s), Set B (30s, 144s, 208s, 1034s), Set C (91s, 509s, 1722s, 10497s). Red indicates oil, blue indicates primary water flooding, and green indicates secondary water flooding.

Taking all these factors into account, the bacterial injection period was set at 30 days Figure 12 shows the original images from the oil displacement experiments, where it is evident that the recovery factor of secondary water flooding (Figure 12C) significantly improved after the microbial huff-and-puff process.

Figure 12A illustrates the primary water flooding process, characterized by a fingering phenomenon. Once the injection channel was fully established, the flooded area no longer expanded, and the sweep efficiency was limited mainly to both sides of the main channel. Figure 12B shows the microbial flooding process, where the bacterial suspension primarily displaced along the main streamline, leaving significant unswept regions on both sides of the channel. Figure 12C depicts the secondary water flooding after 30 days of bacterial injection and incubation. Even after the main flow path was fully injected, the sweep efficiency continued to expand outward. The expanded sweep range was attributed to two key mechanisms: (1) the emulsification and viscosity-reduction effects of bacteria, and (2) the plugging of high-permeability channels by bacterial clusters and biofilms, which altered the pressure distribution within the channels. Through these mechanisms, residual oil outside the main channels could be mobilized effectively, ultimately improving the oil recovery factor.

**FIGURE 13:**
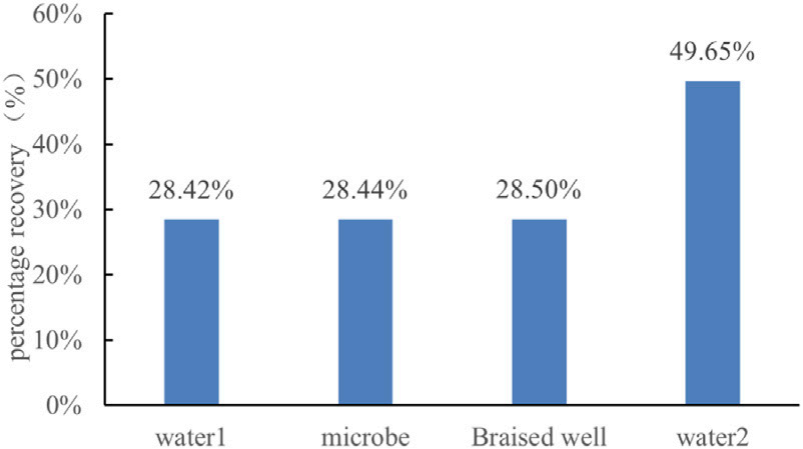
Recovery rate change diagram.

Recovery factor calculations for different displacement stages showed that after the huff-and-puff treatment, the recovery factor of secondary water flooding increased from 28.44% to 49.65%, representing a remarkable 21.21% improvement (Figure 13). Similarly, Liu et al. (2023) employed visual microfluidic devices to displace residual oil using a surfactant (5 wt% Span-80 in brine), achieving a recovery factor of 47.35%. This indicates that microbial oil recovery can, in some cases, achieve results comparable to those of surfactant flooding.

## 4 Conclusion

This study, using microbial visual reservoir chip simulations and oil displacement experiments, revealed the growth patterns, spatial distribution characteristics, and enhanced oil recovery mechanisms of microorganisms at the pore scale, providing essential parameters to support the field application of microbial oil recovery technology. The main conclusions are as follows:

1. Bacterial growth patterns and shut-in time optimization: The growth curve of WJ-8 bacteria followed the Logistic model, with a rapid growth phase lasting 1–3 days (log phase). After 3 days, the bacteria entered the stationary phase and secreted large amounts of metabolites (e.g., biosurfactants). Fitting results showed that bacteria required ≥22.89 h to adapt to reservoir conditions (lag phase), and a shut-in period of ≥2.71 day was necessary to reach the high metabolic activity phase. Considering environmental factors (salinity, pH, temperature) and microbial diffusion and growth conditions, it is recommended that the field shut-in time be extended to 7–30 days to match formation conditions and fully activate metabolic activity.
2. Bacterial spatial distribution and oil displacement mechanism: Bacterial clusters tended to form at blind ends and throat edges due to low flow velocities and abundant attachment points. At blind ends, bacteria aggregated into clusters, while at throat edges, they developed into large bacterial clusters that significantly enhanced biofilm stability and oil detachment ability. Additionally, bacterial metabolites provided synergistic effects: biosurfactants secreted by bacteria reduced interfacial tension, and biofilm plugging of high-permeability zones expanded the sweep area, collectively improving the mobility of residual oil.
3. Bacterial diffusion coefficient: The experimentally calculated bacterial growth diffusion coefficient was 5.0 × 10^-9^–3.0 × 10^−8^ m^2^/s, much higher than that of surfactants (∼10^–10^ m^2^/s). Diffusion rates were lower near the injection point and increased with distance. It required ≥8.14 days for bacteria to diffuse from the main channel to the terminal branches.
4. Enhanced oil recovery: Microbial oil displacement experiments confirmed that after a 30-day shut-in period, the recovery factor of secondary water flooding increased by 21.21% (from 28.44% to 49.65%). The key mechanisms were bacterial emulsification and viscosity reduction, as well as bacterial cluster plugging of high-permeability channels, which altered the pressure distribution in the pores and mobilized residual oil.

Although this study leverages *in situ* observation techniques to advance understanding of microbial growth mechanisms and dynamics at the pore scale in enhanced oil recovery, several limitations remain:

1. Limitations of the microfluidic model: While microfluidic chips effectively replicate reservoir pore structures at the geometric level, they cannot fully capture the complexity of real reservoirs. Microbial behavior in actual reservoirs is influenced by a combination of factors, including mineral composition, temperature gradients, and pressure conditions, which cannot be completely reproduced in laboratory microfluidic setups. As a result, direct application of pore-scale observations to field-scale operations remains challenging. At the reservoir scale, fluid flow dynamics and heterogeneity are more complex, and microbial transport, biofilm formation, and plugging behavior may differ from observations at the pore scale.
2. Limitations of microbial strains and environmental conditions: This study focuses on a single microbial strain under controlled laboratory conditions. Different strains under varying environmental conditions may exhibit significant differences in growth, transport, and oil displacement efficiency, which limits the generalizability of the results to other strains and reservoir conditions.
3. Simplified reservoir conditions: The experimental conditions were simplified, including constant temperature (60 °C) and fixed flow rates, without incorporating key features of actual reservoirs, such as heterogeneity of pore distributions, temperature gradients along the reservoir, variations in salinity during water flooding, and complex multiphase interactions among fluids, rocks, and microbes. In real reservoirs, these factors affect microbial diversity (e.g., dominant strain succession), microbial activity (e.g., metabolic rate regulation), and ultimately oil recovery. Furthermore, reservoir microbial communities are composed of multiple groups, including bacteria, fungi, archaea, and viruses, which interact dynamically over time and space. These interactions produce variable metabolites that alter pore wettability (hydrophilic/hydrophobic transitions) and oil displacement efficiency. The current microfluidic model does not capture these dynamic variables, making it difficult to predict microbial behavior and oil recovery under actual reservoir conditions.
4. Long-term stability and environmental risks: This study primarily focuses on short- and medium-term microbial activity and oil displacement. Long-term stability, microbial survival, potential plugging issues, and the effects of sustained microbial injection and biofilm formation on reservoir permeability were not assessed.

## Data Availability

The original contributions presented in the study are included in the article/supplementary material, further inquiries can be directed to the corresponding authors.
